# Association Between Physical Fitness and Quality of Life in Children and Adolescents with Down Syndrome

**DOI:** 10.3390/children12121611

**Published:** 2025-11-26

**Authors:** Osama Aljuhani, Shomokh Alharbi, Raghad Hakami, Deema Alsaeedi, Wejdan Alqadheb, Najla Al-sadaawi, Fouz Alosaimi

**Affiliations:** 1 Department of Physical Education, College of Sport Sciences and Physical Activity, King Saud University, P. O. Box 2454, Riyadh 11451, Saudi Arabia; 442203447@student.ksu.edu.sa; 2 Department of Exercise Physiology, College of Sport Sciences and Physical Activity, King Saud University, P. O. Box 2454, Riyadh 11451, Saudi Arabia; 446200849@student.ksu.edu.sa (S.A.); 446200540@student.ksu.edu.sa (R.H.); 446200866@student.ksu.edu.sa (D.A.); 446200682@student.ksu.edu.sa (W.A.); 446200733@student.ksu.edu.sa (N.A.-s.)

**Keywords:** down syndrome, quality of life, physical fitness, cardiorespiratory fitness, muscular power, muscular strength

## Abstract

**Background/Objectives**: Multiple studies have reported that the quality of life (QoL) among children and adolescents with Down syndrome (DS) is lower than that among typically developing children. Meanwhile, physical fitness is crucial for improving QoL throughout childhood and adolescence. Combining these insights, this cross-sectional study aimed to examine the association between physical fitness and QoL in children and adolescents with DS. **Methods**: Participants were 56 children and adolescents (aged 8–16 years) enrolled in one DS center in Saudi Arabia. Cardiorespiratory fitness, muscular strength, and muscular power were measured to assess physical fitness. The PedsQL^TM^ (parents’ proxy scale) was used to measure participants’ QoL. Multiple linear regression with pooled analysis was used to examine the association between physical fitness and QoL. **Results**: Boys showed higher muscular power than girls (*p* = 0.024). Weak to moderate correlations were observed between muscular power, muscular strength, and QoL domains. Regression results suggested that children and adolescents with high muscular power had better scores in social functioning (*B* = 0.693, R^2^ = 0.22, *p* = 0.013). Children and adolescents with high muscular strength had better scores in overall QoL (*B* = 2.665, R^2^ = 0.21, *p* = 0.027). **Conclusions**: Children and adolescents with DS who have higher muscular fitness had better scores in social functioning and overall QoL. Increasing muscular fitness in children and adolescents with DS appears to be particularly important for improving QoL.

## 1. Introduction

Down Syndrome (DS)—also known as trisomy 21—is a common condition that strongly impacts children’s daily activities [1]. DS is the most common chromosomal disorder in Saudi Arabia, with an incidence of about 1 per 554 live births [2,3]. Children and adolescents with DS often experience multiple comorbidities such as short stature; obesity; hypothyroidism; sleep disorders; congenital, cardiac, and gastrointestinal anomalies; and musculoskeletal and ophthalmological disorders [4,5,6]. Nevertheless, due to advances in health care, the life expectancy of children with DS has improved over recent decades [7,8]. A previous study suggested that the prevalence of DS will continue to increase in Saudi Arabia due to increasing life expectancy and lower mortality rates [9]. Hence, growing attention has been paid to the quality of life (QoL) in children and adolescents with DS [10].

The World Health Organization (WHO) defines QoL as the way in which individuals perceive their position in life in relation to their culture, values, goals, and standards [11]. Based on the WHO definition, QoL in children and adolescents with DS reflects their ability to meet their needs despite their physical, emotional, and social limitations [12]. Measuring QoL in children and adolescents may thus provide comprehensive insights into how chronic conditions such as DS impact functioning in daily life. Previous studies have consistently reported that QoL in children with DS is lower than QoL in typically developing children. For instance, a recent systematic review and meta-analysis showed that total QoL scores were lower in children with DS compared to typically developing children [13]. Cross-sectional studies have also shown that Saudi children with DS had lower scores in social functioning, school functioning, physical functioning, and total QoL than children without DS [14,15].

Aspects of physical fitness such as cardiorespiratory fitness (CRF) and muscular fitness have been recognized as key health markers in children and adolescents [16]. Furthermore, physical fitness helps with the performance of daily activities; insufficient muscular strength and endurance may restrict independent living in individuals with intellectual disabilities [17,18]. Notably, children and adolescents with DS were reported to have lower levels of physical fitness than their typically developing peers [19,20]. These relatively low levels may be caused by medical conditions such as muscle hypotonia, chronotropic incompetence, motor and developmental delays, and poor postural control [21,22]. Among Saudi children with DS, higher adiposity was found to be significantly associated with lower fitness [23]. Importantly, evidence has shown that physical activity programs improve physical fitness in children and adolescents with DS. For instance, previous intervention studies reported an increase in health-related physical fitness in Spanish adolescents with DS after a 16-week physical activity program comprising three 60 min sessions per week [24,25].

Physical fitness is crucial for improving QoL throughout childhood and adulthood [26,27,28]. Among typically developing children, several studies have reported that physical fitness improves QoL and well-being. For example, in the US, a cross-sectional study reported that CRF and muscular fitness were positively associated with health-related QoL among children aged 9–11 years [26]. Another study indicated that young children with higher levels of physical fitness reported better scores in health-related QoL and well-being than children with low levels of physical fitness [29]. A systematic review and meta-analysis suggested that CRF and muscular fitness are positively associated with health-related QoL in healthy children and adolescents aged under 18 years [30]. However, the link between physical fitness and QoL in children and adolescents with DS has received less attention.

While a few studies have reported a weak association between CRF and some domains of QoL in children and adolescents with DS [14,31], the association between muscular fitness (e.g., muscular strength and power) has not—to the best of our knowledge—been investigated in this population. Identifying this association is crucial for health policy-makers, practitioners, physicians, and school principals. Therefore, this study aimed to examine the association between physical fitness (including muscular strength and power) and QoL domains in children and adolescents with DS in Saudi Arabia. We hypothesized that high levels of CRF and muscular fitness would significantly predict high scores in overall and each domain of QoL.

## 2. Materials and Methods

### 2.1. Participants

This cross-sectional study included 56 children and adolescents with DS (33 boys and 23 girls), aged from 8 to 16 years. The participants were recruited using convenience sampling; they all attended one DS center in Riyadh, Saudi Arabia. The required sample size was calculated using G*Power (version 3.1.9.4). Based on previously established procedures, we determined the sample size based on a medium effect size (0.25), with alpha error probability (0.05), statistical power (0.80), and four predictors. The analysis showed that at least 53 participants were required. A consent form explaining the purpose of the study was distributed in the DS center and signed by parents. The inclusion criteria were: (a) having a diagnosis of mild to moderate DS, (b) aged 8–16 years, (c) able to perform fitness tests. Exclusion criteria were: (a) one or more other major health conditions affecting mobility such as orthopedic or neurological conditions, (b) uncorrected sensory impairments, and (c) congenital heart disease. The study was approved by the King Saud University Ethical Review Committee, Saudi Arabia (ethics code: KSU-HE-25-639, dated: 7 May 2025). All data were collected between May and June 2025.

### 2.2. Measurements

#### 2.2.1. QoL

The participants’ QoL was measured using the Pediatric Quality of Life Inventory (PedsQL^TM^) 4.0 (Arabic version; parents’ proxy scale) [32]. Good validity and reliability were reported in healthy populations and in children with chronic diseases and acute health conditions [32,33]. The scale includes four domains: physical functioning (8 items), emotional functioning (5 items), social functioning (5 items), and school functioning (5 items) [34]. The PedsQL^TM^ was originally designed to measure health-related QoL among healthy children and those with chronic medical conditions [33]. This instrument has previously been used to measure QoL among Saudi children with DS [14,15]. Each item was rated on a five-point rating scale (from 0, “strongly disagree,” to 4, “strongly agree”). Total scores (the sum of all items) range from 0 to 100 points, with 100 indicating the best quality of life. To calculate the score for each domain, we divided the sum of all the scores by the number of items the participants responded to.

#### 2.2.2. Muscular Strength

Muscular strength was measured using the Takei handgrip dynamometry 5401 (Takei Scientific Instruments Co. Ltd., Tokyo, Japan). This measure was previously used among children and adolescents with DS [22,23,35]. When required, the dynamometry was adjusted to fit each participant’s hand size and ensure a comfortable grip. All measurements were performed with participants in a standing position; the wrist was in position with the elbow fully extended. Each participant performed two trials using their dominant hand; the highest score for strength (kg) in the two trials was recorded. A third trial was performed if the value of the second trial was much higher than that of the first; then, the highest value of the second and third trials was recorded.

#### 2.2.3. Muscular Power

Explosive lower-body muscular strength was measured using the standing long-jump test, which has previously been used among children and adolescents with DS [20,22]. Participants were required to jump as far as possible from a standing position, taking-off and landing with their feet together; the distance between take-off and landing point was measured to calculate jump performance. The participants performed two trials, and the best score was used.

#### 2.2.4. Cardiorespiratory Fitness

We used the six-minute walking test (6-MWT) to measure participants’ CRF; this is a standardized test recommended by the American College of Sport Medicine (ACSM) to evaluate cardiorespiratory endurance in populations expected to have low fitness [36]. This test showed good test–retest reliability in people with DS [37] and has previously been used on children and adolescents with DS [14,31]. Participants were required to walk as far as possible within six minutes; the walking distance in meters was recorded.

#### 2.2.5. Anthropometry

Participants’ heights were measured to the nearest ±0.1 cm using a Seca 213 Portable Stadiometer (Hamburg, Germany); body mass was measured to the nearest ±0.1 kg using a Digital Scale (model 770; Seca, Hamburg, Germany). All measurements were conducted and recorded individually while the participants were barefoot and wore light clothing. Body mass index (BMI) was calculated as weight (kg)/height^2^ (m^2^), and converted to z-scores using the LMS method [38].

### 2.3. Data Analysis

There were some missing data (muscular strength: 12 participants, 21.4%; muscular power: 14 participants, 25%; 6-MWT: 9 participants, 16%). Little’s MCAR test was not significant (χ2 = 44.95, df = 47, *p* = 0.558), suggesting that values were missing completely at random. Therefore, all data, including dependent variables, covariates, and predictor variables, were imputed using multiple-imputation techniques in R (4.5.1) with the package *mice* (3.18.0). We constructed 50 imputed datasets with 50 iterations using fully conditional specification-predictive mean matching. This method produces good results for small sample sizes [39]. All results were averaged over imputed datasets using Rubin’s rule [40].

Descriptive statistics were computed as mean and standard deviation (±SD) for normally distributed data and median and interquartile range for skewed data. Frequency and proportion (%) were also used to compute categorial variables. To examine gender differences in QoL and physical fitness variables, the independent *t*-test and Mann–Whitney *U* test were employed according to data normality. Chi-squared test was used to assess differences for categorical data. Effect sizes were reported for each test. To examine the relationship between QoL domains and physical fitness components, we used Pearson correlations and Spearman’s rank correlation. Correlations were considered negligible (0.00 to 0.10), weak (0.10 to 0.39), moderate (0.40 to 0.69), and strong (≥0.70) [41]. We used multiple linear regression with the enter method to examine the association between physical fitness components (predictors) and QoL domains (outcomes). Initial models were created, and then each model was adjusted for age, gender, and BMI z-scores. Previous literature reported potential influences of these covariates on both physical fitness and QoL [22,31,42,43]. We checked all models to ensure that assumptions were not violated. The initial analysis showed that the residuals were normally distributed. We found no multicollinearity among the predictors and covariates (all variance inflation factors < 5). The variance of the residuals across all predictors was constant, indicating homoscedasticity (all *p* > 0.05). Significance level was set as α < 0.05. All analyses were performed using R open-source software (v. 4.5.1, www.r-project.org).

## 3. Results

### 3.1. Characteristics of the Study Sample

Table 1 presents the descriptive statistics for study participants stratified by gender. The results showed that 42 (75%) of the participants were classified as overweight or obese. Compared to boys, girls were significantly older, while boys were significantly heavier (all *p* < 0.05). There were no significant gender differences in family income, schooltime, and degree of DS (all *p* > 0.05).

### 3.2. Children and Adolescents QoL and Physical Fitness

Table 2 displays the mean overall and domain scores for QoL by gender. School functioning had the highest scores in both boys (72.4 ± 17.8) and girls (70.6 ± 16.0), while the lowest mean reported was social functioning for both boys (67.4 ± 22.6) and girls (57.6 ± 26.5). On average, overall QoL for boys was (70.3 ± 14.1), while for girls it was (59.6 ± 12.1). Boys had higher overall scores and in each domain than girls, though these differences were not statistically significant (all *p* > 0.05). As for fitness, boys had higher levels of muscular power than girls (*p* = 0.024, Cohen’s d = 0.80). No significant gender differences were found in either muscular strength or CRF (all *p* > 0.05).

### 3.3. Correlations

Correlations between each domain and muscular fitness are presented in Table 3. We found weak correlations between muscular strength and physical health (r = 0.328, *p* = 0.0244) and overall QoL (r = 0.311, *p* = 0.032). Weak correlations were found between muscular power and physical health (r = 0.352, *p* = 0.015), social functioning (r = 0.384, *p* = 0.007), school functioning (r = 0.324, *p* = 0.025), total QoL (r = 0.439, *p* = 0.001), and muscular strength (r = 0.291, *p* = 0.045). No correlations were found between CRF and QoL domains.

### 3.4. Regression Analysis

Table 4 shows the results of the multiple linear regression models for the association between physical fitness and overall QoL and each domain. In the unadjusted model (model 1), we found that muscular strength was positively associated with physical health (B = 3.240, R^2^ = 0.11), emotional functioning (B = 2.620, R^2^ = 0.10), social functioning (B = 4.110, R^2^ = 0.12), and overall QoL (B = 3.032, R^2^ = 0.16). The results of model 1 also showed that muscular power was significantly and positively associated with social functioning (B = 0.728, R^2^ = 0.18) and overall QoL (B = 0.397, R^2^ = 0.14). When we controlled for age, gender, and BMI z-scores in model 2, muscular strength significantly predicted overall QoL (B = 2.665, R^2^ = 0.21), while muscular power significantly predicted social functioning (B = 0.693, R^2^ = 0.22). No associations were found between CRF and the QoL domains (all *p* > 0.05)

## 4. Discussion

Our study is among the first to examine the association between physical fitness and QoL in children and adolescents with DS, as well as looking at gender differences in this regard. Our results indicated that both measures of muscular fitness (muscular power and strength) were significantly and positively correlated with QoL domains. Regression results demonstrated that muscular power significantly predicted social functioning. There was also evidence that muscular strength significantly predicted total QoL. No association was found between CRF and QoL domains.

### 4.1. Overall Levels of QoL and Physical Fitness

Generally, we found a favorable level of QoL in both overall scores and in each domain. These results agree with a previous Saudi study, which showed similar levels in all domains of QoL (66.5–69.7) among children with DS [14]. Our results also support another Saudi study, which reported a similar level of QoL (71.9–75.2) in all domains [15]. Furthermore, a similar online survey study including 211 parents from 18 countries showed that parents reported moderate levels in all domains of QoL for children and adolescents with DS [44]. In our study, consistent with previous Saudi studies [14,15], the lowest scores were in the social function domain. This is not surprising, given that children with DS have generally weaker social capabilities compared to typically developing children [45]. This result may also reflect the fact that Saudi children and adolescents with DS have fewer interactions with their families and communities. Children and adolescents with DS may also experience challenges such as social isolation, restricted opportunities, and other stress-related factors [46]. More research is needed to understand the influence of family environment, individual skills, and community variables on children and adolescents’ QoL in the Saudi context.

As for physical fitness, our results showed low levels of CRF and muscular fitness among participating children and adolescents with DS. Similarly, a previous review reported relatively low levels of CRF among children and adolescents with DS compared to their typically developing peers [47]. The current results also agreed with a previous study that reported low levels of CRF among Saudi children with DS [14]. Three physiological factors are considered as potential explanations of low levels of CRF in children and adolescents with DS: metabolic dysfunction, autonomic dysfunction, and reduced ventilatory capacity [47]. As for muscular fitness, the low levels observed in the current study can be explained by many factors. For instance, it has been reported that there is a reduction by 50% in upper and lower muscular strength in people with DS compared to typically developing individuals [48,49]. This reduction negatively impacts the performance of daily activities [50]. Low levels of muscular fitness could also be attributed to muscle hypotonia, which is a common characteristic of DS [51]. Our results are in agreement with previous studies, which reported that adolescents with DS had lower levels of muscular fitness compared to typically developing children [20,52]. Therefore, interventions are needed to improve CRF and muscular fitness in children and adolescents with DS.

### 4.2. Gender Differences

Our results showed no gender differences in total QoL scores or in any domain. These results are inconsistent with those of Alrayes et al., who reported that Saudi girls with DS had better levels in school and learning domains than boys [53]; however, direct comparison with these findings is problematic given the difference in measurements of QoL domains. Namely, we used PedsQL, while KIDSCREEN-27 was used in the previous study [53]. A recent review reported that children with DS had significantly lower scores in social and school functioning domains when PedsQL was used compared to KIDSCREEN [13]. Hence, more investigation and development of tools to measure QoL in children and adolescents with DS is needed.

We also observed no significant gender differences in CRF and muscular strength, although boys showed higher muscular power than girls. Both boys and girls in the current study were recruited from one DS center, suggesting that they were engaged in comparable levels of activity during structured exercises. Our results are partly consistent with those of a previous study, which reported no difference in muscular fitness between genders in children with intellectual disabilities [54]. Our results also agreed with those that reported no differences between boys and girls in 44 motor skills, including walking and jumping [55].

### 4.3. Correlation of Fitness with QoL

Our study extends the existing literature by investigating the direct correlation between fitness levels and QoL in children and adolescents with DS. We found that physical fitness was positively correlated with overall QoL and each domain. Specifically, muscular power was significantly correlated with physical health, social functioning, school functioning, and total QoL. Muscular strength was only correlated with physical health and total QoL. These findings can be interpreted within the context of the study’s sample environment. Namely, a possible explanation is that children and adolescents included in this study attended a specialized center that, alongside academic classes, provided structured activity lessons. The participants might be encouraged and supported with opportunities to engage in activities to develop their muscular fitness. It has been reported that structured physical activity appears to be effective in improving muscular strength in children and adolescents with DS [56]. Moreover, recent systematic reviews reported that physical activity intervention programs significantly improved upper and lower body strength in individuals with DS [1,57]. These improvements are crucial for independence and well-being in this population. Therefore, the positive correlation between muscular fitness and QoL observed in this study can be explained by the broad benefits that physical activity offers for QoL. Although direct correlation between muscular strength and QoL has not previously been reported, physical activity interventions aiming to increase muscular strength may increase autonomy and independence in daily activities, which are important for QoL [57]. Our results are consistent with a similar Egyptian study, which found a moderate correlation (*r* = 0.34, *p* = 0.04) between gross motor skills (i.e., walking, running, and jumping, which are crucial for muscular fitness improvement) and QoL in children with DS [58]. The current results showed that CRF was not correlated with overall QoL and all its domains. These results do not support the previous study that observed a positive correlation between QoL and CRF in young Saudi children (aged 6-12 years) [14]. This discrepancy may be explained as a result of differences in habitual physical activity levels across samples and age groups. of physical activity. However, our novel finding highlights the crucial role of fitness for broader development and QoL. Intervention studies with extended follow-up are needed to evaluate the benefits of muscular fitness on QoL in this population.

### 4.4. Association Between Physical Fitness and QoL

The associations between physical fitness and specific domains of QoL in children and adolescents with DS have not been previously investigated. Notably, we found a positive association between muscular power and social functioning. Our results strongly suggest that improving muscular fitness leads to improvements in social well-being and QoL in children and adolescents with DS, independent of gender, age, and body size. This suggestion supports the results of a previous review, which indicated that physical activity interventions enhance both fitness and QoL in people with DS [57]. This finding also supports Cuesta Vargas et al.’s study relating to adults with intellectual disability, which reported that adults with higher levels of muscular strength had higher self-reported levels of QoL [59]. Our results are also in agreement with a previous Chinese intervention study, which found that an adapted physical activity program significantly improved both handgrip strength and QoL in adolescents with intellectual disability [60].

Meanwhile, the current results showed that CRF was not associated with QoL. These results are inconsistent with a previous study that found higher levels of CRF predicted higher scores in QoL in young children with DS [14]. Discrepancy with the previous study may be due to the lower levels of CRF observed in this study. Furthermore, our use of parent-proxy report could be another explanation, as this method may not have reflected participants’ actual QoL. Nonetheless, our novel findings highlight the crucial role of fitness for broader development and QoL. Intervention studies with extended follow-ups are needed to better evaluate the benefits of muscular fitness on QoL in this population. Furthermore, studies targeting children and adolescents with DS, with a large sample size and controlling for potential covariates such as dietary habits, parental support, physical environment, and motor skills, are warranted to confirm our results.

### 4.5. Practical Implications

This study suggests that promotion of muscular fitness in clinical and educational settings may improve the physical fitness of children and adolescents with DS. Adapted physical activity programs targeting muscular fitness may also be a powerful strategy to improve QoL in this population. In addition to improving fitness, these programs provide opportunities for social and emotional development. Notably, children and adolescents with DS may struggle to follow or misunderstand the instructions during fitness activities. Therefore, validated and reliable assessment tools tailored for children and adolescents with DS are needed for any practical interventions; these would enable accurate monitoring of the different aspects of fitness and evaluation of intervention outcomes. Furthermore, due to the high prevalence of overweight and obesity in this population (as confirmed in this study), which is a risk factor for cardiovascular issues, it is crucial to integrate physical fitness programs with nutritional counseling and multidisciplinary care. Alongside fitness programs, monitoring body composition can guide personalized intervention to lower the cardiometabolic risk.

### 4.6. Limitations

Despite the novel findings outlined above, our results should be interpreted with caution due to the following limitations. The cross-sectional nature of the current study means that causality between muscular fitness and QoL cannot be identified. Longitudinal studies are needed to continue examining the association between muscular fitness and QoL in this population. We did not investigate the influence of potential factors such as quality of sleep and mood, despite previous literature showing that these factors are linked to physical fitness in children [61,62] and adults [63]. Furthermore, our study was conducted in a single DS center; thus, the results should be interpreted with caution. Future research should investigate whether our results are consistent in other settings. Finally, we used convenience sampling, which is prone to selection bias, and QoL was reported by parents, which may also introduce bias.

## 5. Conclusions

Our results show that children and adolescents with DS had moderate levels in overall QoL and in each domain. Low levels of physical fitness were reported. No significant gender differences were found in overall QoL, QoL domains, CRF, and muscular strength. The only significant gender difference was observed in muscular power, where higher levels were found in boys. Weak to moderate correlations were reported between QoL domains and physical fitness. Muscular strength was found to significantly predict overall QoL; muscular power was found to predict social functioning. Thus, increasing muscular fitness in children and adolescents with DS may be particularly important for improving QoL.

## Figures and Tables

**Table 1:** Demographic characteristics of the participants by gender.

	Boys	Girls	*t*	z	*X* ^2^	*p*-Value	ES
	N = 33	N = 23					
**Age (years)**	9.0 (1)	11 (4)		−3.658		<0.001	1.08
**Height (cm)**	126.1 ± 7.5	130.8 ± 10.1	−1.985			0.052	0.53
**Weight (kg)**	33.1 ± 8.7	43.4 ± 14.0	−3.396			0.001	0.92
**BMI (kg·m^−2^)**	24.6 ± 2.6	23.4 ± 2.6	−3.340			0.002	0.90
**BMI z-score**	1.7± 1.6	1.9 ± 1.2	−0.546			0.565	0.14
**Income**					0.582	0.748	0.10
**Low**	15 (45.5%)	11 (47.8%)					
**Moderate**	8 (24.2%)	7 (30.4%)					
**High**	10 (30.3%)	5 (21.7%)					
**School time**					0.471	0.492	0.09
**Morning**	23 (69.7%)	14 (60.9%)					
**Afternoon**	10 (30.3%)	9 (39.1%)					
**DS Degree**					1.371	0.242	0.15
**Mild**	12 (36.4%)	5 (21.7%)					
**Moderate**	21 (63.6%)	18 (78.3%)					

Note: Data are presented as Mean ± SD; Median (interquartile range); Number (%); *t* = *t* test; z = Standardized test; *X*^2^ = Chi square; ES: Effect Size (Cohen’s d for *t*-test, the rank-biserial correlation for Mann–Whitney *U* test, phi coefficient and Cramer’s V for *X*^2^); DS: Down syndrome; BMI: Body mass index.

**Table 2:** Descriptive statistics for study variables according to gender.

	Boys	Girls	*t*	z	*p*-Value	ES
	N = 33	N = 23				
**PH**	69.9 ± 19.1	61.2 ± 20.7	−1.619		0.162	0.43
**EF**	71.3 ± 14.9	63.4 ± 20.6	−1.658		0.163	0.45
**SF**	67.4 ± 22.6	57.6 ± 26.5	−1.485		0.191	0.40
**SCF**	72.4 ± 17.8	70.6 ± 16.0	−0.380		0.705	0.10
**Total QoL**	70.3 ± 14.1	59.6 ± 12.1	−1.670		0.162	0.45
**MS**	6.3 (2.4)	5.5 (1.5)		−2.510	0.225	0.34
**MP**	67.4 (23.7)	55.7 (12.9)		−1.136	0.024	0.80
**CRF**	211.9 ± 92.9	227.4 ± 77.3		0.597	0.589	0.11

Note: Data are presented as Mean ± SD; Median (interquartile range); PH: Physical health; EF: Emotional functioning; SF: Social functioning; SCF: School functioning; MS: Muscular strength; MP: Muscular power; CRF: Cardiorespiratory fitness; QoL: Quality of Life; *t* = *t* test; z = Standardized test; ES: Effect Size (Cohen’s d for *t*-test, the rank-biserial correlation for Mann–Whitney U test).

**Table 3:** Correlations for QoL domains and physical fitness.

	PH	EF	SF	SCF	Total QoL	MS	MP	CRF
**PH**								
**EF**	0.611 **							
**SF**	0.480 **	0.422						
**SCF**	0.638 **	0.381	0.499 **					
**Total QoL**	0.859 **	0.751 **	0.790 **	0.758 **				
**MS**	0.328 *	0.129	0.179	0.209	0.311			
**MP**	0.352 *	0.272	0.384 *	0.324 *	0.439 **	0.291 *		
**CRF**	−0.083	−0.064	−0.014	−0.237	−0.106	0.160	−0.218	

Note: PH: Physical health; EF: Emotional functioning; SF: Social functioning; SCF: School functioning; MS: Muscular strength; MP: Muscular power; CRF: Cardiorespiratory fitness; QoL: Quality of Life; * *p* < 0.05; ** *p* < 0.01.

**Table 4:** Associations between QoL and physical fitness.

		*B*	95% CI	SE	*p*-Value
	Physical Health				
**MS**	Model 1	3.240	0.332, 5.995	1.438	0.030
	Model 2	2.840	−0.188, 5.743	1.438	0.065
**MP**	Model 1	0.369	−0.142, 0.838	0.231	0.121
	Model 2	0.298	−0.230, 0.800	0.243	0.230
**CRF**	Model 1	−0.013	−0.088, 0.067	0.036	0.702
	Model 2	−0.013	−0.086, 0.063	0.035	0.727
	**Emotional functioning**				
**MS**	Model 1	2.620	0.244, 5.090	1.209	0.035
	Model 2	2.604	−0.009, 5.307	1.325	0.056
**MP**	Model 1	0.268	−0.108, 0.654	0.183	0.151
	Model 2	0.192	−0.239, 0.636	0.209	0.365
**CRF**	Model 1	−0.003	−0.060, 0.061	0.029	0.921
	Model 2	0.001	−0.058, 0.066	0.030	0.976
	**Social functioning**				
**MS**	Model 1	4.110	0.500, 7.547	1.734	0.023
	Model 2	3.486	−0.503, 7.297	1.907	0.076
**MP**	Model 1	0.728	0.222, 1.217	0.245	0.005
	Model 2	0.693	0.153, 1.234	0.265	0.013
**CRF**	Model 1	−0.020	−0.109, 0.073	0.044	0.650
	Model 2	−0.021	−0.110, 0.071	0.043	0.626
	**School functioning**				
**MS**	Model 1	2.157	−0.367, 4.544	1.246	0.092
	Model 2	1.730	−0.773, 4.142	1.245	0.173
**MP**	Model 1	0.224	−0.213, 0.641	0.203	0.278
	Model 2	0.237	−0.170, 0.646	0.199	0.242
**CRF**	Model 1	−0.036	−0.094, 0.028	0.029	0.223
	Model 2	−0.042	−0.095, 0.013	0.026	0.140
	**Total QoL**				
**MS**	Model 1	3.033	0.825, 5.147	1.100	0.009
	Model 2	2.665	0.331, 4.922	1.158	0.027
**MP**	Model 1	0.397	0.025, 0.752	0.173	0.029
	Model 2	0.355	−0.021, 0.728	0.180	0.057
**CRF**	Model 1	−0.018	−0.074, 0.044	0.028	0.523
	Model 2	−0.019	−0.073, 0.040	0.027	0.482

Note: Model: Unadjusted model; Model 2: Adjusted for age, gender, and BMI z-scores; MS: Muscular strength; MP: Muscular power; CRF: Cardiorespiratory fitness; QoL: Quality of Life; *B*: Unstandardized beta coefficient; SE: Standard error; CI: Confidence interval.

## Data Availability

All relevant data are available in the manuscript.

## Abbreviations

The following abbreviations are used in this manuscript:

QoL	Quality of life
PH	Physical health
SF	Social functioning
SCF	School functioning
EF	Emotional functioning
CRF	Cardiorespiratory fitness
MS	Muscular strength
MP	Muscular power
ES	Effect size
*t*	*t*-test
z	Standardized test
*X*^2^	Chi square
CI	Confidence interval
